# Supplementary material for: Genetic comparisons of interleukin-17 reveal a framework for complex signaling evolution
Source: bioRxiv. 2026 Apr 14:2026.04.13.718218. Preprint. [Version 1] doi: 10.64898/2026.04.13.718218 (PMC13105070; doi:10.64898/2026.04.13.718218)
Supplement: Supplement 3 [file media-3.zip › Supplemental files for paper/ERC/IL17xIL17_comparisons/IL17RB_IL17RB.pdf]

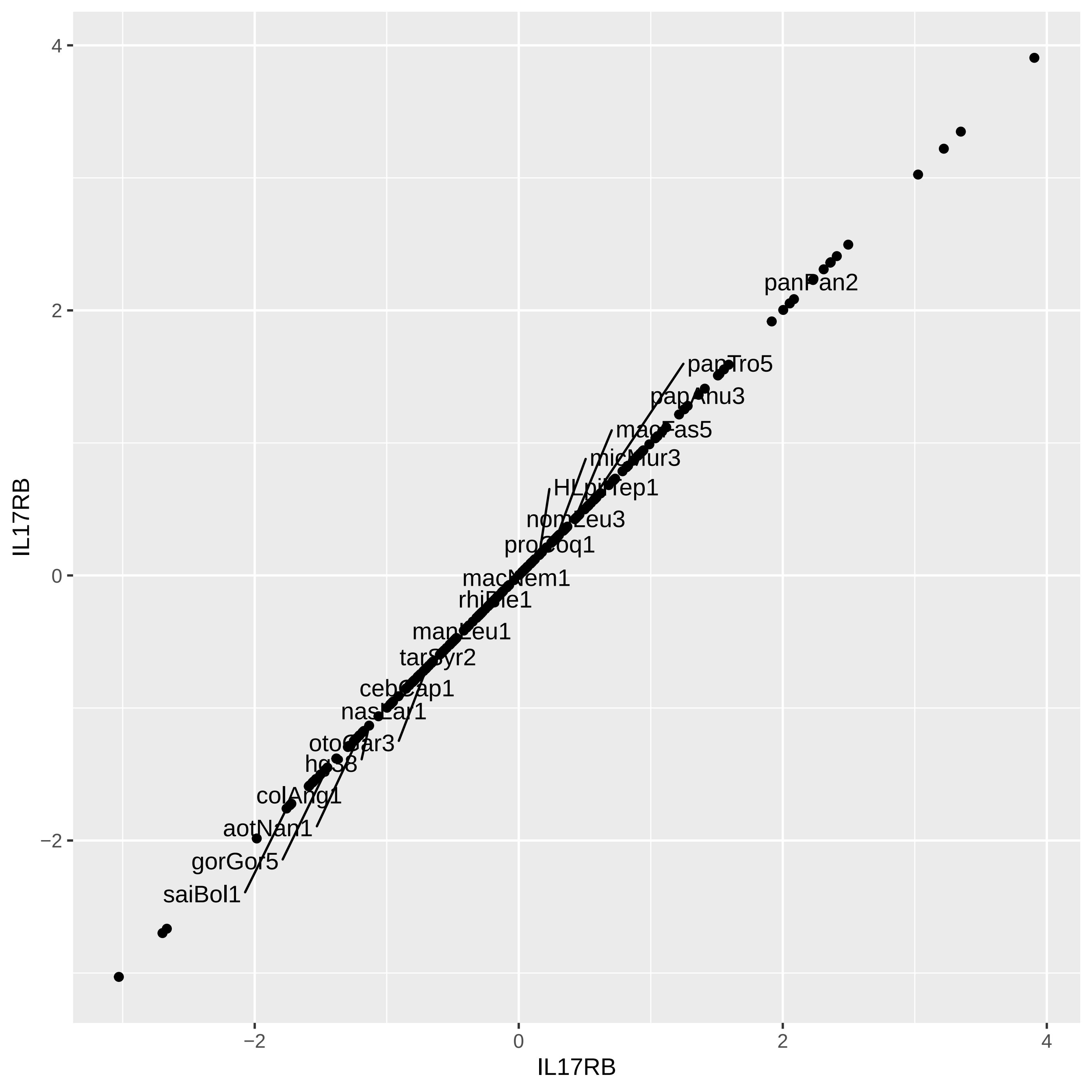
panPan2
panTro5
papAnu3
macFas5
micMur3
HLpilTep1
nomLeu3
proCoq1
macNem1
rhiBie1
manLeu1
tarSyr2
cebCap1
nasLar1
otoGar3
hg38
colAng1
aotNan1
gorGor5
saiBol1
IL17RB
IL17RB
−2
0
2
4
